# Structural, Physical, and Mechanical Analysis of ZnO and TiO_2_ Nanoparticle-Reinforced Self-Adhesive Coating Restorative Material

**DOI:** 10.3390/ma14247507

**Published:** 2021-12-07

**Authors:** Qura Tul Ain Idrees, Nazish Gul, Muhammad Amber Fareed, Salman Aziz Mian, Danish Muzaffar, Muhammad Nasir, Aqif Anwar Chaudhry, Sultan Akhtar, Syed Zubairuddin Ahmed, Abdul Samad Khan

**Affiliations:** 1 Department of Science of Dental Materials, Postgraduate Medical Institute, Lahore 54000, Pakistan; aineshahzad@gmail.com (Q.T.A.I.); gul.nazish029@gmail.com (N.G.); 2 Adult Restorative Dentistry, Dental Biomaterials and Prosthodontics Oman Dental College, Muscat 116, Oman; mafareed@staff.odc.edu.om; 3 Department of Dental Materials, Institute of Dentistry, CMH Lahore Medical College, Lahore 54000, Pakistan; salman_aziz@cmhlahore.edu.pk; 4 Faculty of Dentistry, SEGi University, Petaling Jaya 47810, Malaysia; danish9181@hotmail.com; 5 Interdisciplinary Research Centre in Biomedical Materials, COMSATS University Islamabad, Lahore Campus, Lahore 54000, Pakistan; muhammadnasir@cuilahore.edu.pk (M.N.); aqifanwar@cuilahore.edu.pk (A.A.C.); 6 Department of Biophysics, Institute for Research and Medical Consultations, Imam Abdulrahman Bin Faisal University, Dammam 31441, Saudi Arabia; suakhtar@iau.edu.sa; 7 Department of Restorative Dental Sciences, College of Dentistry, Imam Abdulrahman Bin Faisal University, Dammam 31441, Saudi Arabia; szahmed@iau.edu.sa

**Keywords:** dental materials, EQUIA coat, glass ionomer, FTIR, TGA, DSC, hardness, zinc oxide, titanium dioxide, nanoparticles

## Abstract

This study aimed to modify an EQUIA coat (EC; GC, Japan) by incorporating 1 and 2 wt.% of zinc oxide (ZnO; EC-Z1 and EC-Z2) and titanium dioxide (TiO_2_; EC-T1 and EC-T2) nanoparticles, whereby structural and phase analyses were assessed using Fourier transform infrared spectroscopy (FTIR) and X-ray diffraction (XRD), respectively. Thermogravimetric analysis/differential scanning calorimetry, micro-hardness, and water absorption analyses were conducted, and the microstructure was studied by scanning electron microscopy/energy-dispersive spectroscopy. FTIR spectra showed a reduction in peak heights of amide (1521 cm^−1^) and carbonyl (1716 cm^−1^) groups. XRD showed peaks of ZnO (2θ ~ 31.3°, 34.0°, 35.8°, 47.1°, 56.2°, 62.5°, 67.6°, and 68.7°) and TiO_2_ (2θ ~ 25.3°, 37.8°, 47.9, 54.5°, 62.8°, 69.5°, and 75.1°) corresponding to a hexagonal phase with a wurtzite structure and an anatase phase, respectively. Thermal stability was improved in newly modified materials in comparison to the control group. The sequence of obtained glass transitions was EC-T2 (111 °C), EC-T1 (102 °C), EC-Z2 (98 °C), EC-Z1 (92 °C), and EC-C (90 °C). EC-T2 and EC-T1 showed the highest (43.76 ± 2.78) and lowest (29.58 ± 3.2) micro-hardness values. EC showed the maximum water absorption (1.6%) at day 7 followed by EC-T1 (0.82%) and EC-Z1 (0.61%). These results suggest that EC with ZnO and TiO_2_ nanoparticles has the potential to be used clinically as a coating material.

## 1. Introduction

Glass ionomer cement (GIC) is a material of choice in pediatric dentistry and is a commonly used material in adult dentistry due to the fact of its tendency to bond chemically with enamel/dentin with an added advantage of fluoride release [1,2]. For almost the past 50 years, GICs have been successfully used in dentistry and are available in various modified forms. Resin-modified glass ionomer (RMGIC) and glass carbomer have shown better physical properties compared to conventional GICs [3,4]. GICs can be used as a restorative material in their own right or as a base for a composite resin overlay (sandwich restoration). Conventional GIC was considered unsuitable for posterior restorations (i.e., Class I and II) due to the fact of its poor mechanical performance [5]; however, it showed successful results in Class V restorations [6]. RMGIC has been used for Class II restoration; however, wide variations in success rates have been observed [5]. In 2007, a new high-viscous GIC restorative system (EQUIA; GC Europe, Tokyo, Japan) was introduced and was designed for use in the permanent restoration of Class I, II, and V cavities [7]. A recent study showed that both GI- and resin-based composite restorations showed good survival after 10 years, and no significant differences were observed in the clinical performances when used for Class I and II restorations [8]. 

GICs are hydrolytically unstable in their early stages if exposed to moisture, and the leaching of ions occurs and dehydrates if exposed to air during the first hour of mixing. Therefore, isolation from water contamination should be maintained for at least 24 h [9] during the different phases of precipitation, gelation, and hydration of GICs. Otherwise, a rough surface allows plaque accumulation and harbors bacterial colonization leading to increased risk of secondary caries, poor mechanical properties, and decreased esthetics [10,11]. 

To overcome the problem of moisture contamination, a surface coating over the restoration has been recommended [12]. For surface protection of GICs, different surface protecting agents have been applied after initial setting reactions such as Cavitine^®^, copal varnish (SS White, Rio de Janeiro-RJ, Brazil), Magic^®^ Bond Adhesive (Vigodent, Rio de Janeiro-RJ, Brazil), solid petroleum gel, and nail varnish [13]. The nano-filled and self-adhesive resin coating, G-Coat PLUS, provides a perfect seal of a GIC surface [14,15]. The resin coat binds micro-mechanically and interlocks with the GIC and stays for a long time on the surface of GICs. Therefore, during the complete setting reaction of GIC, the surface protection is maintained, which results in increased wear resistance and flexural strength of the restoration material [16,17]. An EQUIA coat based on nanotechnology exhibited improved physical and mechanical properties of GI restoration compared to other conventional coatings [18]. The EQUIA system (GC, Tokyo, Japan) is a combination of a chemically cured, highly filled GIC (Fuji IX GP Extra, GC, Tokyo, Japan), which is self-adhesive and a light-cured, filled resin surface sealant (G-Coat PLUS, GC, Tokyo, Japan). The compressive, flexural, and diametral strength of the EQUIA system and ChemFil Rock were statistically significantly higher compared to Fuji IX Gold Label and Ketac Molar Easymix [19], having better marginal adaptation and a higher retention rate [12]. Moreover, an EQUIA coat is recommended for Class I and smaller Class II cavities as a permanent restoration material [20]. Infiltration of a protective coating, such as an EQUIA Coat on GIC, provides internal protection, and its protective effect from extrinsic water may also allow for the complete maturation of the GIC restoration with delayed water contact [21].

The main cause of restoration failure in dentistry is secondary caries; however, to overcome this problem and to prolong restoration life, metallic nanoparticles with antimicrobial properties are incorporated into restorative materials [22]. Several types of nanoparticles, such as zinc oxide (ZnO), titanium dioxide (TiO_2_), silver, gold, and copper, have been incorporated in dental restoratives to impart antibacterial properties [23]. Among metal oxides, significant inhibitory and antimicrobial effects have been shown by TiO_2_ and ZnO nanoparticles [24]. GIC reinforced with TiO_2_ nanoparticles has shown antibacterial activity against *Streptococcus mutans* (*S. mutans*) without interfering with the release of fluoride in GICs and has been used successfully in high stress-bearing areas to endure the forces of mastication [25]. Likewise, incorporation of ZnO nanoparticles into the powder of Fuji IX at a 3 wt.% concentration showed a significant increase in the antibacterial property of set GIC [26].

Therefore, it would be advantageous to formulate a novel coating material for GIC by incorporation of TiO_2_ and ZnO nanoparticles that would possess the desirable properties of both preventions from moisture contamination along with enhanced mechanical and antibacterial properties. Thus, the objectives of the present study were to incorporate nanoparticles of ZnO and TiO_2_ into a commercially available GIC coating material (EQUIA coat, GC Tokyo, Japan) followed by the investigation of structural, thermal, mechanical, and physical properties of the resultant product. The null hypothesis was that the addition of ZnO and TiO_2_ nanoparticles into the EQUIA coat will not affect the mechanical, thermal, and morphological properties of the EQUIA coat.

## 2. Materials and Methods

EQUIA coat (Lot no. 1407041) was purchased from GC Tokyo, Japan, and was used as it was received. According to the manufacturer, EQUIA coat (EC) contains methyl methacrylate, urethane methacrylate, camphorquinone, and phosphate ester monomer. ZnO (200–500 nm) and TiO_2_ (40–70 nm) were synthesized by our group through precipitation and the sol-gel hydrothermal method, respectively, as described previously [26,27]. The SEM images of both TiO_2_ and ZnO are provided in the Supplementary Materials (Figure S1). Acetone (Lot no. 320110, Sigma-Aldrich, St. Louis, MO, USA) was used as a solvent.

### 2.1. Sample Preparation 

To prepare experimental samples, ZnO and TiO_2_ nanoparticles were incorporated into the EC. For one batch preparation, 1 mL (1.298 g) of EC was dissolved in 0.5 mL acetone (CAS #666-52-4, Sigma-Aldrich, St. Louis, MO, USA) at room temperature under continuous stirring at 100 rpm using a magnetic stirrer (Wiggens, Straubenhardt, Germany), followed by the addition of 1 wt.% (0.0129 g) and 2 wt.% (0.0259 g) nanoparticles of ZnO and titania in increments under continuous stirring at 150 rpm for 30 min to evaporate the acetone. The disc-shaped (5 mm × 2 mm) specimens of the experimental group (Table 1) were prepared by pouring the material in prefabricated molds, and the specimens were covered with a mylar strip after placement in the mold and cured for 60 s on both sides with a light-cured LED curing unit (Woodpecker, Shanghai, China) with a 395–480 nm wavelength and an irradiance level of 800 mv/cm^2^. After complete curing, the disc-shaped specimens were carefully retrieved from the molds and polished smoothly on both sides with 800, 1200, 1500, and 4000 grit-sized papers (3M Flexible Polishing papers, Maplewood, MN, USA).

### 2.2. Fourier Transform Infrared (FTIR) Spectroscopy 

FTIR spectroscopy with attenuated total reflectance (ATR) (Thermo Nicolet 6700, Thermo Scientific, Waltham, MA, USA) was used and a background scan was obtained before each set of tests. FTIR spectra of five polymerized specimens from each group (Table 1) were measured by placing them on the diamond window of FTIR. The spectra were collected over the region 4000–400 cm^−1^ at a resolution of 8 cm^−1^, averaging 256 scans. The data were analyzed using OMINIC v9.2 software (Thermo Fischer Scientific, Waltham, MA, USA) and peaks were matched with the literature.

### 2.3. X-ray Diffraction (XRD) Analysis 

XRD analysis was performed at room temperature on X’pert powder PAN analytical diffractometer (Malvern Panalytical, Malvern, UK) with a copper (Cu) X-ray source (Malvern Panalytical, Malvern, UK). at 40 kV voltage and 30 mA current source. Samples were ground (with pestle and mortar) to form the powder. Samples were scanned between 2θ = 10°–90° with a step size of 2θ = 0.02. XRD analysis assists in the assessment and quantification of the crystalline or amorphous structure of samples related by Bragg’s law to measure inner atomic spaces through an angle of reflection:

Nλ = 2d.sinθ(1)

where λ is the wavelength in nm, n is an integer, d is the inner spacing, and theta (θ) is the angle diffraction. 

### 2.4. Scanning Electron Microscopy (SEM)

SEM was performed to measure the surface morphology, size of filler, and filler distribution of the control and experimental samples with an SEM machine (TESCAN VEGA-3 LMU, Brno, Czech Republic) with an energy-dispersive X-ray (EDX) analytical system (Oxford Instruments, Abingdon, UK). Specimens were gold-coated in a gold sputter coater (QUORUM Technologies, Lewes, UK) with a voltage of 20 kV and a magnification of 1500–5000× to study the nanostructure.

### 2.5. Thermal Gravimetric Analysis/Differential Scanning Calorimetric Analysis (TGA/DSC)

The glass transition temperature (Tg) and initial degradation temperature of the specimens were measured at a temperature range of 25–800 °C with a heating rate of 10 °C/min, with simultaneous TGA/DSC (TA Instrument: SDT Q600 V8.0 Build95, New Castle, DE, USA) under an inert nitrogen atmosphere with a flow rate of 100 mL/min. Sample discs were cut into small pieces, and approximately 15 mg of each sample was taken to measure decomposition temperature ranges. Thermal analysis (TG 2 (SF), Mettler Toledo, Greifensee, Switzerland) was also conducted under a continuous flow of air (25 mL/min) within a temperature range of 25–800 °C and with a heating rate of 10 °C/min. The derivative weight loss was analyzed.

### 2.6. Vickers Micro-Hardness (VH) Test

Six disc-shaped, polished specimens of each group were tested with an HVS-100 source (Digital Micro Vickers Hardness Tester, Beijing, China), testing range of 1 HV–2967 HV, dwell time of 10 s, and test forces of 100 gf. The specimens were subjected to three indentations at different points, the diagonal length of the specimens was measured, and the hardness was calculated according to the following formula:

VH = 1.85 P/d^2^ (2)

where VH denotes the Vickers hardness number, P denotes the indentation load, and d denotes the indentation diagonal.

### 2.7. Water Sorption Analysis

Water absorption analysis was carried out according to the method described in ANSI/ADA Specification No. 27-2016 (ISO 4049-2009). The samples (5 × 2 mm) (*n* = 6) were weighed and recorded as initial weight, *W_i_*. Then, all samples were immersed in 5 mL deionized water at 37 °C for 1 and 7 days. The samples were removed periodically from deionized water, blotted dry, and transferred to a drying oven (DAIHAN Scientific Co., Ltd., Seoul, Korea) maintained at 37 °C for 2 h. The samples were again weighed until the equilibrium was maintained and calculated as *W_f_*. After each interval, samples were immersed in fresh solution and stored again at 37 °C in an incubator source (DAIHAN Scientific Co., Ltd., Seoul, Korea) and the percentage weight for each specimen was calculated by the following formula:

Water sorption % = (*W_f_* − *W_i_*/*W_i_*) × 100 (3)

### 2.8. Statistical Analysis 

The data were analyzed by SPSS version 20 (IBM Corp, Armonk, NY, USA). The mean ± standard deviation (SD) was calculated for quantitative variables, whereas frequency and percentages were calculated for qualitative variables. The normality of quantitative variables was determined using the Shapiro–Wilk test. The mean difference between groups was compared by applying one-way ANOVA and post hoc tests. Chi-square or Fisher’s exact test were used to observe the association of qualitative variables with the groups. A *p*-value ≤ 0.05 was considered statistically significant.

## 3. Results

### 3.1. Fourier Transform Infrared Spectroscopy

**Figure 1:**
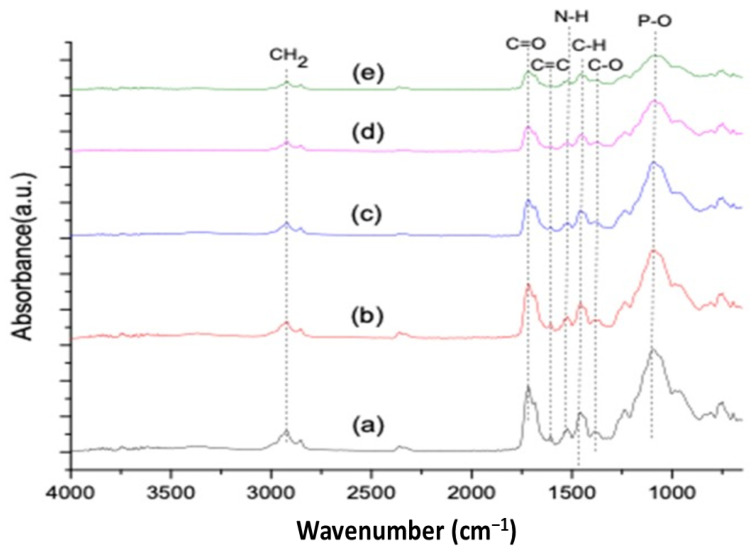
Comparative FTIR spectra of (**a**) EC-C, (**b**) EC-T1, (**c**) EC-T2, (**d**) EC-Z1, and (**e**) EC-Z2.

The FTIR spectra of the control and experimental samples are shown in Figure 1, and the peak intensities are tabulated in Table 2. The EC-C spectrum showed a carbonyl stretch peak at 1711 cm^−1^ in resin coat, while an aromatic ring of benzene (C=C) was present in resin matrices at 1606 cm^−1^. The N–H deformation stretching of urethane dimethacrylate appeared at 1509 cm^−1^. A weak peak at 1034 cm^−1^ showed asymmetric stretching of C–O–C and Si–O stretching vibration, indicating the presence of silicates. After incorporation of titania nanoparticles (1 and 2 wt.%) into EC, a different IR spectrum was observed and a significant decrease in peak intensities can be identified in Figure 1. The peak at 1711 cm^−1^ corresponded to the free carbonyl group, and the intensity decreased (EC-C~0.38, EC-T1~0.31, and EC-T2~0.22) which is attributed to the consumption of free carbonyl during interactions with titania nanoparticles. The methacrylate resin showed a peak of the aromatic C=C group at 1606 cm^−1^. The resins carrying aromatic rings also lessened in intensity (EC-C~0.09, EC-T1~0.08, and EC-T2~0.08) which contributed to the reaction of C=C with titania and conversion of C=C to C–C. The peak of N–H attributed to the deformation of UDMA at 1509 cm^−1^ also showed a low intensity (EC-C~0.14, EC-T1~0.13, and EC-T2~0.10) after the addition of titania nanoparticles. FTIR spectra comparison showed a reduction in peak heights of methacrylate, carbonyl, urethane, and other groups that was greater in EC-T2 compared to EC-T1 as shown in Table 2. 

Similarly, FTIR spectra having characteristic peaks of EC-Z1 and EC-Z2 are shown in Figure 1, indicating that after incorporation of ZnO nanoparticles (1 and 2 wt.%) into EC, a different IR spectrum was obtained and a significant decrease in peak intensities was observed. The peak at 1711 cm^−1^ corresponded to the free carbonyl group and lessened in intensity in EC-Z1 and EC-Z2 compared to EC-C (EC-C~0.38, EC-Z1~0.22, and EC-Z2~0.11), which contributed to the consumption of free carbonyl during interactions with ZnO nanoparticles. The aromatic C=C group of the methacrylate resin corresponded to the peak at 1606 cm^−1^. The benzene ring of resins also lessened in intensity (EC-C~0.09, EC-Z1~0.03, and EC-Z2~0.03), which showed the conversion of C=C to C–C. The peak of N–H, attributed to the deformation of urethane dimethacrylate at 1509 cm^−1^, also showed a low intensity (EC-C~0.14, EC-Z1~0.05, and EC-Z2~0.04). The comparative FTIR spectra of all experimental samples showed the same positions; however, a difference was observed in the peak heights of the carbonyl, methacrylate, urethane, and phosphate groups in the following order: EC-C > EC-T1 > EC-T2 > EC-Z1 > EC-Z2 as shown in Table 2.

### 3.2. X-ray Diffraction Analysis 

**Figure 2:**
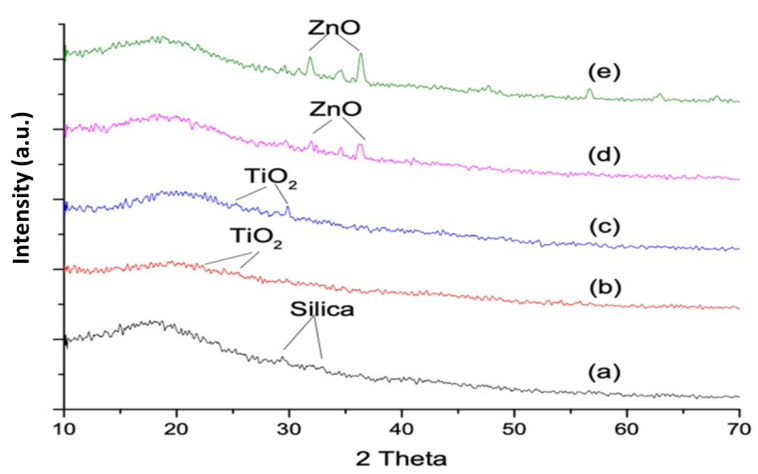
XRD characteristic metallic peaks located in the range of 2θ~20–75°: (**a**) EC-C, (**b**) EC-T1, (**c**) EC-T2, (**d**) EC-Z1, and (**e**) EC-Z2.

A comparison of the XRD pattern of control and experimental groups is shown in Figure 2. Characteristic metallic peaks of TiO_2_ were in the range of 2θ~20–75°. X-ray diffraction measurements showed six distinct diffraction peaks of EC-T1 and EC-T2 at 25.3°, 37.8°, 47.9°, 54.5°, 62.8°, 69.5°, and 75.1°. Nanoparticles of TiO_2_ exhibited a crystalline nature with peaks lying at 2θ ≈ 25.25° (101), 37.8° (004), 47.9° (200), 53.59° (105), and 62.36° (204) as shown in Figure 2b,c. The orientation plane (101) was observed in both EC-T1 and EC-T2. A diffractogram showed EC-T2 at 25.3° and 32° compared to EC-T1. All the peaks in the XRD diffraction data were in agreement with JCPDS file #21-1272 [28].

The characteristic peak of ZnO is given in Figure 2d,e at scattering angles (2θ) of 31.3°, 34.0°, 35.8°, 47.1°, 56.2°, 62.5°, 67.6°, and 68.7°, corresponding to the reflection of the 100, 002, 101,102, 110, 103, 200, and 112 crystal planes, respectively. The XRD pattern of ZnO corresponded to the hexagonal phase with a wurtzite structure as per the standard JCPDS specification pattern for ZnO (file #043-0002).

### 3.3. Scanning Electron Microscopy

**Figure 3:**
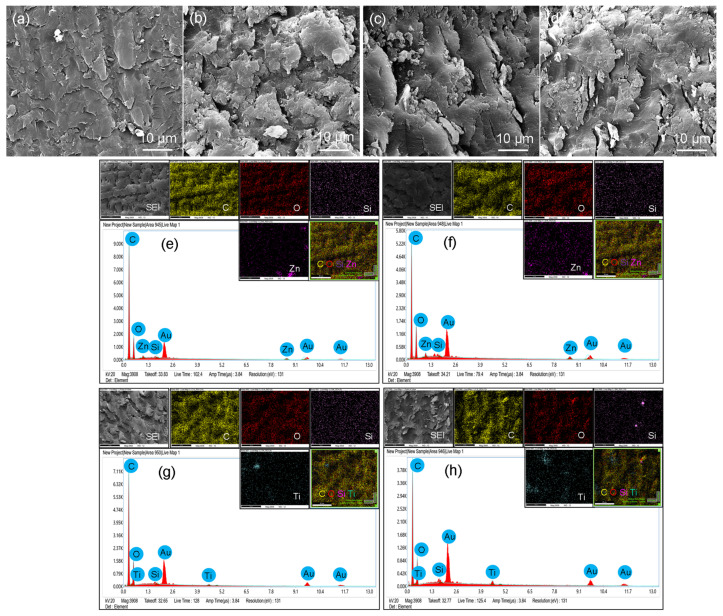
Comparison of SEM images: (**a**) EC-Z1, (**b**) EC-Z2, (**c**) EC-T1, and (**d**) EC-T2. EDS spectra and EDX mapping of (**e**) EC-Z1, (**f**) EC-Z2, (**g**) EC-T1, and (**h**) EC-T2. The EDS spectra confirmed the presence of Zn and Ti in EC. The gold peak appeared due to the gold-coating of the samples. The EDS mapping images show the distribution of ZnO and TiO_2_ nanoparticles in EC. The scale bars are 10 µm (**a**–**d**) and 20 µm (**e**–**h**) for the mapping images.

SEM images, EDS spectra, and EDS mapping of EC-Z1, EC-Z2, EC-T1, and EC-T2 are represented in Figure 3. The SEM images of EC-Z1, EC-Z2, EC-T1, and EC-T2 show the distribution of ZnO and TiO_2_ fillers and EC meshwork resin (Figure 3a–d). Titania nanoparticles were spherical and globular in shape with a 70–100 nm average particle size, while ZnO nanoparticles were randomly distributed by an average of 200–500 nm with elongated and rod-like shapes placed haphazardly and transversely. EDS spectra of both the composites showed the presence of Zn in corresponding EC specimens (EC-Z1 and EC-Z2) and Ti in EC-T1 and EC-T2 (Figure 3e–h). As expected, the intensity of the Zn peak was higher for EC-Z2 than EC-Z1 due to the higher concentration of Zn in EC. Similar results were found for EC-T1 and EC-T2, where the Ti level was higher for the 2% TiO_2_ than the 1% TiO_2_ nanoparticle specimen. Moreover, the EDS mappings were further confirmed in the presence of Zn and Ti and their uniform distribution throughout the EC network resin.

### 3.4. Thermogravimetric Analysis and Differential Scanning Calorimetry

**Figure 4:**
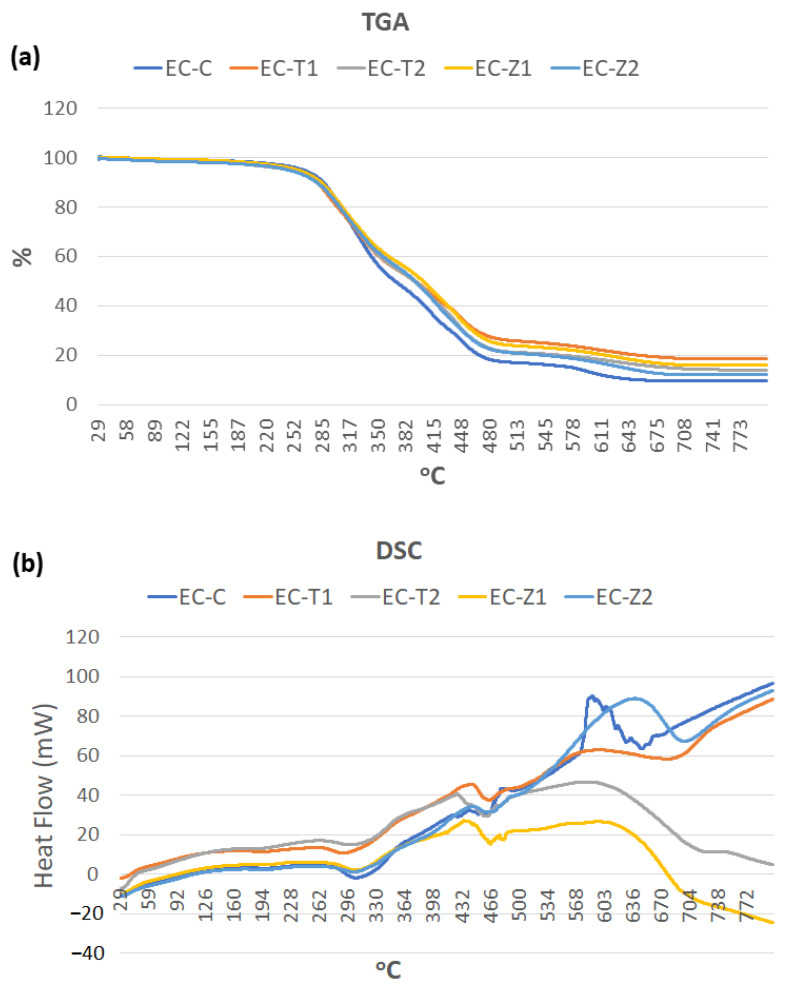
(**a**) TGA and (**b**) DSC graphs showing the degradation in the specimens with an EQUIA coat and the experimental groups with temperature change.

Figure 4a shows the percentage of samples changing with an increase in the temperature in the TGA (under inert nitrogen) of all cured samples up to 800 °C. The degradation occurred in three steps. There was a negligible loss of mass till 100 °C, as there was no water present in the specimens. At approximately 300 °C, a considerable loss of mass (almost 20%) was detected; it was credited to the volatilization of smaller molecular weight components (i.e., camphorquinone), whereas another rapid loss in mass (70%) was observed after 400 °C due to the burning of larger molecular weight (i.e., UDMA and PMMA) organic components in the specimens. At 600 °C, all organic constituents were burned, and inorganic components remained. From 600 °C, no significant loss in mass was found, and it was assigned to the absence of the polymer network or cross-links in the unpolymerized specimens that decreased the stability of the sample. At 600 °C, EC-C showed 90% weight loss, whereas EC-T1, EC-Z1, EC-Z2, and EC-T2 showed 83%, 81%, 80%, and 78% weight loss, respectively, indicating that the increased concentration of ZnO and titania nanoparticles resulted in the reduced weight loss of specimens. The EC-Z2 showed the lowest weight % change, while EC showed the highest weight loss at 450 °C. The % weight loss with increasing temperature is shown in Table 3, where the observed sequence of % weight loss was EC-C > EC-T1 > EC-Z1 > EC-Z2 > EC-T2. 

The DSC showed (Figure 4b) that the thermal profile, such as glass transition (Tg) and the initial degradation temperature (IDT), of different events occurred due to the heating of the composite groups. UDMA containing dental polymers indicated that the corresponding polymers had a Tg of approximately 90–119 °C, depending on the number of monomers and polymerization time. The sharp endothermic and exothermic peaks showed a broadening of peaks after the addition of nanoparticles. Tg was in the order: EC-T2 (111 °C) > EC-T1 (102 °C) > EC-Z2 (98 °C) > EC-Z1 (92 °C) > EC-C (90 °C), whereas the initial degradation temperature was in the order: EC-Z2 > EC-Z1 > EC-T1 > EC-T2 > EC-C (Table 3). An almost similar trend was observed when the thermal analysis was conducted under air, where samples with high concentrations (i.e., 2 wt.%) showed thermal stability. However, under air, EC-T2 showed the relatively lowest weight % change (20.5%) compared to EC-Z2 (18.2%), EC-Z1 (16.9%), EC-T1 (17%), and EC-C (15.6%). The T_onest_, T_max_, and residue at T_max_ of each group are given in Table 4 and the derivative thermal loss is presented in Figure S2a–e.

### 3.5. Vickers Micro-Hardness Test

**Figure 5:**
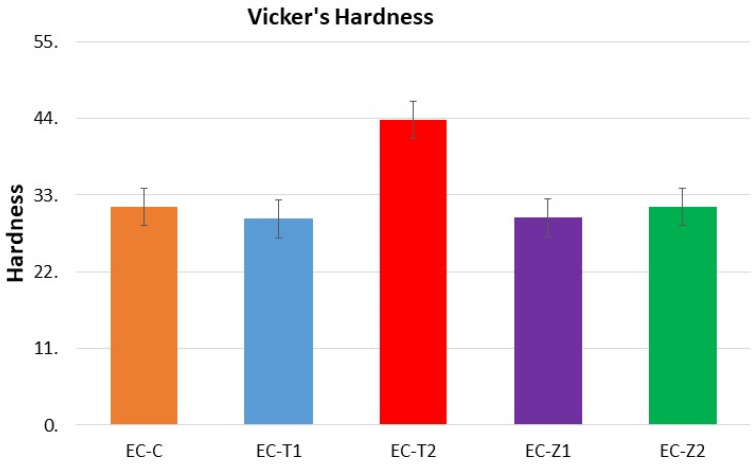
Vickers micro-hardness of the EQUIA-coated and experimental groups.

The comparative means and standard deviation values of the micro-hardness of the composite groups are shown in Figure 5. The observed sequence was EC-T2 (43.76 ± 2.78) > EC-C (31.33 ± 0.92) > EC-Z2 (30.18 ± 2.35) > EC-Z1 (29.71 ± 2.09) > EC-T1 (29.58 ± 3.23). The statistical analysis revealed a significant difference between all groups with a *p*-value ≤ 0.05. There was a significant difference (*p* = 0.000) between EC-C and EC-T2 as well as EC-T1 (*p* = 0.00). EC-T2 had a significant difference with all groups at 0.00 values. EC-Z1 had a significant difference with EC-T2 at 0.00 values, and EC-Z2 had a significant difference with EC-T2 at 0.00 values. The results show that EC-T2 had the highest Vickers micro-hardness and EC-T1 exhibited the lowest. The normality of the data was assessed by the Shapiro–Wilk test, and the results revealed that the data were normally distributed.

### 3.6. Water Sorption Analysis

**Figure 6:**
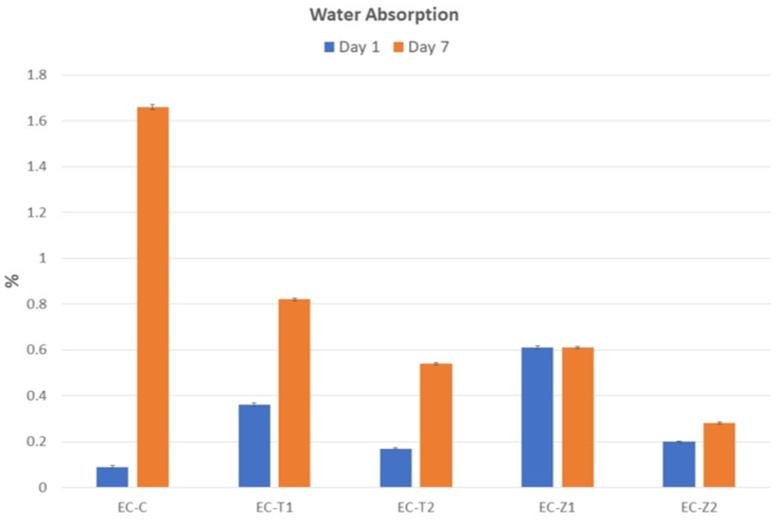
The mean (SD) water absorption values of the control and experimental groups at days 1 and 7.

It was found that water absorption decreased with the increased incorporation of nanoparticles. Measurements of weight change after days 1 and 7 of water immersion are given in Figure 6. The statistical analysis showed that the mean weight change percentage was due to the fact of water sorption in the EC-Z1 group, which was significantly higher compared to the remaining groups. Whereas no significant difference was observed in the remaining groups. 

On day 7, for multiple comparisons, the post hoc Tukey test showed that the mean weight change percentage due to the fact of water absorption in the EC-C group was significantly higher compared to the remaining groups, whereas the mean weight change percentage of the EC-Z2 group was significantly lower compared to all the remaining groups. No significant difference was observed among the EC-T1, EC-T2, and EC-Z1 groups.

## 4. Discussion

This study was based on the preparation and comparative analysis of the mechanical, thermal, morphological, and physical properties of the EQUIA coat with and without reinforcement of TiO_2_ and ZnO nanoparticles in different weight percentages. The modified materials showed comparable properties with the control group positively. All the properties and results attained in this study are in accordance with the achievement of the objective. The Fourier transform infrared spectrum of the resin composite showed an intense stretching peak at 1730 cm^−1^ corresponding to carbonyl groups (C=O), an amide N–H absorption band at 1537 cm^−1^ was observed due to the presence of urethane dimethacrylates, and the peak at 1400–1450 cm^−1^ represented the aliphatic C–H vibrations from the resin matrix [29]. Si–O was located at 1300–1250 cm^−1^ which indicated the presence of SiO_2_ filler [30]. It was observed that the peak heights of the carbonyl, methacrylate, urethane, and phosphate groups decreased considerably with an increase in the concentration of nanoparticles. The peak heights of the carbonyl (0.38), methacrylate (0.12), urethane (0.14), and phosphate (0.58) groups in EC-C were higher than all other experimental groups. There was a remarkable reduction in the peak heights of carbonyl C=O, aromatic C=C, and amide N–H in the EC-Z1 and EC-Z2 groups compared to EC-T1 and EC-T2. The change in peak heights could be due the presence of either an accumulation of these functional groups or due to the interactions of functional groups with nanoparticles (i.e., ZnO and TiO_2_). Nanosized ZnO reacted spontaneously with acid groups of the liquid and formed zinc methacrylate or dimethacrylate, which contributed to cross-linking reactions [31]. ZnO nanoparticles might interact with the urethane group through hydrogen bonds (O–H) with the carbonyl group; therefore, a reduction in the band intensity of the N–H group occurred as a result of hydrogen bond formation between ZnO and the urethane group [32]. 

TiO_2_ nanoparticles interacted with urethane through the OH group, creating a polar bond as observed by the N–H stretching zones at approximately 1528 and 3380 cm^−1^. It is reported that the TiO_2_ nanoparticles can establish polar linkages through OH groups with polar polymer (e.g., urethane) more effectively than that of the non-polar polymer (e.g., polypropylene and polyethylene) [32].

X-ray diffraction analysis was used to identify the amorphous or crystalline structure of the material [33], and the diffraction peaks located at 31.84°, 34.52°, and 36.33° were keenly indexed as the hexagonal wurtzite phase of ZnO and related to the diffraction planes of hexagonal ZnO (PDF #75–1526) at (1 0 0), (0 0 2), (1 0 1), respectively [34]. In EC-Z2 more and prominent diffraction peaks were located for the ZnO nanorods compared to EC-Z1. A rod-like ZnO nanostructure was found to be randomly distributed in the EQUIA coat. TiO_2_ diffraction peaks with values lying at 2θ = 29.50 corresponded to the anatase phase and were confirmed by the International Centre for Diffraction Data (ICDD Ref: 98-009-6946) [35]. The anatase phase of the TiO_2_ nanoparticles showed high photocatalytic activity including killing bacteria, fungi, and viruses and preventing recurrent caries and enamel demineralization [36]. These metal oxides (i.e., ZnO and TiO_2_) possess antibacterial properties, inhibit bacterial growth, and might help prevent secondary caries [37]. The dental restorative materials without antibacterial agents may lead to more accumulation of plaque around restoration margins than other restorative materials carrying antibacterial agents [38]. TiO_2_ nanoparticle-reinforced dental resins showed significant antimicrobial effects and resulted in the prevention of recurrent caries and enamel demineralization [36]. However, the inhibition of biofilm formation within the oral cavity through nanoparticles possessing antimicrobial effects has been incorporated into polymeric materials as a function of their biocidal effect [39].

SEM showed that nanoparticles of ZnO exhibited varying morphologies such as flower-like, rod-like, and grains. The ZnO nanorods controlled the formation of biofilms, which reduced the chances of secondary caries [1]. In the present study, 200–500 nm sized ZnO nanorods were incorporated into the EQUIA coat to inhibit secondary caries and improve mechanical strength. However, hardness was not improved after the addition of ZnO nanorods. In contrast, the TiO_2_ group revealed that the TiO_2_ nanoparticles were spherical and distributed inside the matrix. The surface morphology of the TiO_2_ nanoparticles exhibited a higher degree of integrity and smooth surface [40]. It was reported that a resin-based composite containing 10% TiO_2_ nanoparticles had a significant effect on the reduction of colony counts for Streptococcus *mutans* and Streptococcus *sanguinis* [41]. In this study, initially, the concentration of nanoparticles was from 0.5 to 10 wt.%. It was found that high-intensity light failed to penetrate through the nanoparticles at 3 wt.% and was unable to cure the resin completely (evaluated by FTIR spectroscopy). Therefore, 2 wt.% was considered as the maximum concentration of filler component in the EQUIA coat. 

According to the results of the TGA, it was found that the greater the concentration of inorganic nanoparticles, the greater the thermal stability. The steep curve observed between 400 and 460 °C was due to the breakdown of the dental composite backbone (PMMA and UDMA) [42]. The second step at 452 °C may have been due to the degradation temperature of PMMA. PMMA first decomposed at 290 °C, corresponding to the monomer formation at the end unit containing unsaturated bonds. The main decomposition process, which occurred at 390 and 400 °C, was attributed to the degradation of the polymer backbone (PMMA and UDMA) [43]. UDMA showed two degradation steps with a maximum rate of 357 and 444 °C [44]. The greater the percentage of polymer backbone (MMA) (i.e., EC-C), the lower the thermal stability. After the complete breakdown of the polymer matrix at 700 °C, only the inorganic nanoparticles were left. The onset of the degradation temperature increased slightly, and complete deterioration occurred at a higher temperature. It was illustrated in a study that after incorporation of TiO_2_ nanoparticles in a composite, thermal stability improved as the TGA test demonstrated that the decomposition temperature increased. It was previously reported that the incorporation of nanoparticles at higher temperatures could improve the thermal stability of polymer composites [45]. Variations in results were observed when the samples were tested under the continuous flow of nitrogen gas and air. The onset of temperature increased when the samples were run under air. The initial decomposition under an air atmosphere occurred at a higher temperature than in nitrogen. However, the final decomposition of samples treated under an air atmosphere occurred at a lower temperature compared to nitrogen gas. The delay in the initial decomposition might have been due to the interaction of dimethacrylate resins with oxygen, and the results are in accordance with previously reported data [46]. The samples under both nitrogen and air showed thermal stability, and a negligible change in weight loss was observed up to 100 °C. A previous study reported that the maximum mouth temperatures with hot fluids can rise to approximately 70 °C [47]; therefore, it is expected that the samples analyzed in the current study are suitable for clinical applications. The DSC curve of UDMA-containing composites showed a glass transition in the range of 58–130 °C [48]. Intraoral temperatures that exceed the glass transition of restorative material may lead to material softening and, subsequently, in the failure of the clinical restorations. In polymeric materials, the glass transition temperature is a very important phenomenon, as it determines the physical state and final mechanical properties of the material. The glass transition of all experimental and control groups was higher than the intraoral temperature range, i.e., 0–70 °C. Very hot liquids can raise the intraoral temperature to 70 °C, and the consumption of iced drinks can lower it to approximately 0 °C for the front teeth [47]. Within a practically relevant temperature range between 20 and 60 °C, materials such as porcelain and glass ionomer types of cement are well adapted to the tooth structure; however, resinous composites expand more than the tooth tissue [49]. 

The degradation and durability of dental materials are evaluated by surface hardness tests [12]. All control and experimental samples used in this study were finished and polished before testing. It was reported that the finishing and polishing of a dental restorative material’s surface increased the values of micro-hardness compared to unfinished and unpolished surfaces [50]. The application of nanotechnology in dental resin composites was developed to improve the mechanical strength and enhance the antimicrobial properties effectively [1]. The hardness value recorded for the EC-T2 was the highest (43.76 ± 2.78), and the lowest was for EC-T1 (29.58 ± 3.23). The highest value of hardness recorded in the present study was (43.76 ± 2.78) for EC-T2, and it can be considered to bear masticatory forces very efficiently. This increased value of EC-T2 compared to the remaining study groups could be due to the presence of spherical titania nanoparticles. However, rod-like ZnO nanorods did not show any enhancement in the mechanical strength of the material. ZnO has been used more frequently in dental materials due to the fact of its antibacterial properties and reduced risk of secondary caries and plaque accumulation around the margins of restorations. 

The material investigated in the present study consisted of UDMA and PMMA. Highly hydrophilic monomers (i.e., methacrylate) after the uptake of water could exceed the polymerization shrinkage value. The organic phase in filler could additionally increase the water sorption. This could be explained by the high values of stress reduction due to the water sorption results in hygroscopic relaxation [51]. The addition of nanoparticles in resin composites caused a substantial reduction in the contact angle of composites. However, the water contact angle increased, and the wettability of nanoparticles was reduced in water. The greater the percentage of nanoparticles, the lesser the wettability and water sorption (hydrophobicity) compared to resin composites without nanoparticles [52]. In this study, water uptake was due to the resin matrix and was high in EC-C. Components from a resin-based material (short-chain polymers and residual monomers, metallic ions) can elute into oral salivary fluids which can interact with mucosal tissue or can also diffuse towards dentine and pulpal tissue. Weight losses of up to 2% of the mass of the composite have been reported [53]. However, a biocompatibility study was not conducted and is considered as a limitation of this current study.

## 5. Conclusions

Within the limitations of this study, it was concluded that the addition of ZnO and TiO_2_ nanoparticles in the EQUIA coat improved the physical and mechanical properties. The influence of both nanoparticles in different concentrations was evident.

- The spectral changes were observed at the carbonyl region (1711 cm^−1^), and the deformation of the N–H peak was observed at 1521 cm^−1^. The change in peak intensity was more so in the ZnO-based groups compared to the TiO_2_-based samples;
- The SEM/EDS confirmed the uniform distribution of samples, whereby the XRD pattern of the ZnO-based samples showed characteristic peaks corresponding to the hexagonal phase with a wurtzite structure, and the anatase phase was confirmed for the TiO_2_-based samples;
- The TGA and DSC curves showed that the thermal decomposition and glass transition temperature increased with higher concentrations of nanoparticles, indicating a strong molecular interaction of organic–inorganic components. The sequence of weight loss (%) was EC-C > EC-T1 > EC-Z1 > EC-Z2 > EC-T2, whereby the initial degradation temperature was in the order: EC-Z2 > EC-Z1 > EC-T1 > EC-T2 > EC-C. The observed sequence for Tg was above the range of the intraoral temperature;
- The increase in the concentration of spherical TiO_2_ nanoparticles resulted in increased micro-hardness values contrary to the micro-hardness of the ZnO-nanoparticle-reinforced material. Experimental adhesive with a 2 wt.% of TiO_2_ showed the highest hardness values compared to all other groups, and the 1 wt.% of the TiO_2_-based samples showed the lowest hardness values;
- The control group (i.e., EQUIA coat) showed the maximum water absorption after 7 days. The ZnO 2 wt.%-based samples showed the lowest water absorption value;
- Future studies may also evaluate the in vitro biological (microbiological and cytotoxicity) performances of these experimental materials in addition to the detailed mechanical testing and elution analysis of organic/inorganic components.

## Figures and Tables

**Table 1:** Distribution of the control and experimental groups.

Sr.	Groups	wt.% of Nanoparticles
1	EC-C	EQUIA coat-Control
2	EC-T1	TiO_2_ 1 wt.% + EC
3	EC-T2	TiO_2_ 2 wt.% + EC
4	EC-Z1	ZnO 1 wt.% + EC
5	EC-Z2	ZnO 2 wt.% + EC

**Table 2:** Comparison of the FTIR pattern of EC-C with EC-T1, EC-T2, EC-Z1, and EC-Z2.

Wavenumber (cm^−1^)	Assigned Groups	Absorbance (Intensity)				
		EC-C	EC-T1	EC-T2	EC-Z1	EC-Z2
2923	CH_2_	0.12	0.11	0.10	0.06	0.03
1716	C=O	0.38	0.31	0.22	0.22	0.11
1606	C=C	0.09	0.08	0.08	0.03	0.03
1521	N–H	0.14	0.13	0.10	0.05	0.04
1457	C–H	0.22	0.21	0.18	0.09	0.09
1386	C–O	0.11	0.11	0.11	0.09	0.05
1050	C–O–C	0.58	0.49	0.44	0.29	0.19

**Table 3:** Thermal weight percentage loss values (under inert nitrogen gas) in the specimens with an EQUIA coat and the experimental groups.

Groups	TGA (Weight Loss Percentage)					DSC (Thermal Profile)	
	100 °C	300 °C	350 °C	450 °C	600 °C	Tg	IDT
EC-C	1%	22%	42%	77.6%	90%	90 °C	276.5 °C
EC-T 1	1%	19%	40%	73%	83%	102 °C	278.3 °C
EC-T 2	1%	18%	40%	66%	78%	111 °C	277 °C
EC-Z 1	1%	17.3%	36%	69%	81%	92 °C	280.5 °C
EC-Z 2	0.8%	16.8%	36.8%	68%	80%	98 °C	282.8 °C

**Table 4:** The onset and maximum temperature and residual presence of the control and experimental groups.

	EC-T1	EC-T2	EC-Z1	EC-Z2	EC
T_onset_	365.19 °C	342.65 °C	328.21 °C	346.28 °C	365 °C
T_max_	443.63 °C	450.73 °C	452.09 °C	450.85 °C	446.43 °C
Residue at T_max_	17%	20.50%	16.90%	18.20%	15.60%
Final Residue	1.55%	4.10%	1.93%	2.01%	0.05%

## Data Availability

Data is contained within the article or Supplementary Materials.

## Supplementary Materials

The supplementary files are available online at https://www.mdpi.com/article/10.3390/ma14247507/s1, Figure S1: SEM images of (a) titanium dioxide and (b) zinc oxide nanoparticles, where the particle size of titanium dioxide and zinc oxide are 50–100 nm and 200–500 nm, respectively; Figure S2: (a) Thermogram of EC-C showing weight loss (%) and derivative weight loss; (b) Thermogram of EC-T1 showing weight loss (%) and derivative weight loss; (c) Thermogram of EC-T2 showing weight loss (%) and derivative weight loss; (d) Thermogram of EC-Z1 showing weight loss (%) and derivative weight loss; (e) Thermogram of EC-Z2 showing weight loss (%) and derivative weight loss.

## References

[1] Ahmed K.E., Murbay S. (2016). Survival rates of anterior composites in managing tooth wear: Systematic review. J. Oral Rehabil..

[2] Fuhrmann D., Murchison D., Whipple S., Vandewalle K. (2020). Properties of new glass-ionomer restorative systems marketed for stress-bearing areas. Oper. Dent..

[3] Nicholson J.W., Sidhu S.K., Czarnecka B. (2020). Enhancing the mechanical properties of glass-ionomer dental cements: A review. Materials.

[4] Moshaverinia M., Navas A., Jahedmanesh N., Shah K.C., Moshaverinia A., Ansari S. (2019). Comparative evaluation of the physical properties of a reinforced glass ionomer dental restorative material. J. Prosthet. Dent..

[5] Chadwick B.L., Evans D.J.P. (2007). Restoration of class II cavities in primary molar teeth with conventional and resin modified glass ionomer cements: A systematic review of the literature. Eur. Arch. Paediatr. Dent..

[6] Francisconi L.F., Scaffa P.M.C., de Barros V.R.D.S.P., Coutinho M., Francisconi P.A.S. (2009). Glass ionomer cements and their role in the restoration of non-carious cervical lesions. J. Appl. Oral Sci..

[7] Brkanović S., Ivanišević A., Miletić I., Mezdić D., Jukić Krmek S. (2021). Effect of Nano-Filled Protective Coating and Different pH Enviroment on Wear Resistance of New Glass Hybrid Restorative Material. Materials.

[8] Hutchison C., Cave V. (2019). 10 year comparison of glass ionomer and composite resin restoration materials in class 1 and 2 cavities. Evid. Based Dent..

[9] Tyagi S., Thomas A.M., Sinnappah-Kang N.D. (2020). A comparative evaluation of resin-and varnish-based surface protective agents on glass ionomer cement—A spectrophotometric analysis. Biomater. Investig. Dent..

[10] Ong J.E.-X., Yap A., Hong J.Y., Eweis A.H., Yahya N.A. (2018). Viscoelastic properties of contemporary bulk-fill restoratives: A dynamic-mechanical analysis. Oper. Dent..

[11] Najeeb S., Khurshid Z., Zafar M.S., Khan A.S., Zohaib S., Martí J.M.N., Sauro S., Matinlinna J.P., Rehman I.U. (2016). Modifications in glass ionomer cements: Nano-sized fillers and bioactive nanoceramics. Int. J. Mol. Sci..

[12] Brito C.R., Velasco L.G., Bonini G.A., Imparato J.C.P., Raggio D.P. (2010). Glass ionomer cement hardness after different materials for surface protection. J. Biomed. Mater. Res. Off. J. Soc. Biomater. Jpn. Soc. Biomater. Aust. Soc. Biomater. Korean Soc. Biomater..

[13] Gurgan S., Kutuk Z.B., Ergin E., Oztas S.S., Cakir F.Y. (2017). Clinical performance of a glass ionomer restorative system: A 6-year evaluation. Clin. Oral Investig..

[14] Gurgan S., Kutuk Z., Ergin E., Oztas S., Cakir F. (2015). Four-year randomized clinical trial to evaluate the clinical performance of a glass ionomer restorative system. Oper. Dent..

[15] Kielbassa A.M., Oehme E.P., Shakavets N., Wolgin M. (2021). In vitro wear of (resin-coated) high-viscosity glass ionomer cements and glass hybrid restorative systems. J. Dent..

[16] Kanik Ö., Turkun L.S., Dasch W. (2017). In vitro abrasion of resin-coated highly viscous glass ionomer cements: A confocal laser scanning microscopy study. Clin. Oral Investig..

[17] Hesse D., Bonifácio C.C., Kleverlaan C.J., Raggio D.P. (2018). Clinical wear of approximal glass ionomer restorations protected with a nanofilled self-adhesive light-cured protective coating. J. Appl. Oral Sci..

[18] Molina G.F., Cabral R.J., Mazzola I., Lascano L.B., Frencken J.E. (2013). Mechanical performance of encapsulated restorative glass-ionomer cements for use with Atraumatic Restorative Treatment (ART). J. Appl. Oral Sci..

[19] Friedl K., Hiller K.-A., Friedl K.-H. (2011). Clinical performance of a new glass ionomer based restoration system: A retrospective cohort study. Dent. Mater..

[20] Bagheri R., Taha N., Azar M., Burrow M. (2013). Effect of G-Coat Plus on the mechanical properties of glass-ionomer cements. Aust. Dent. J..

[21] Hamouda I.M. (2012). Current perspectives of nanoparticles in medical and dental biomaterials. J. Biomed. Res..

[22] Farrugia C., Camilleri J. (2015). Antimicrobial properties of conventional restorative filling materials and advances in antimicrobial properties of composite resins and glass ionomer cements—A literature review. Dent. Mater..

[23] Lee J.K., Choi J.Y., Lim B.S., Lee Y.K., Sakaguchi R.L. (2004). Change of properties during storage of a UDMA/TEGDMA dental resin. J. Biomed. Mater. Res. Off. J. Soc. Biomater. Jpn. Soc. Biomater. Aust. Soc. Biomater. Korean Soc. Biomater..

[24] Garcia-Contreras R., Scougall-Vilchis R.J., Contreras-Bulnes R., Sakagami H., Morales-Luckie R.A., Nakajima H. (2015). Mechanical, antibacterial and bond strength properties of nano-titanium-enriched glass ionomer cement. J. Appl. Oral Sci..

[25] Vanajassun P.P., Nivedhitha M., Nishad N., Soman D. (2014). Effects of zinc oxide nanoparticles in combination with conventional glass ionomer cement: In vitro study. Adv. Hum. Biol..

[26] Nasir M., Rauf S., Muhammad N., Nawaz M.H., Chaudhry A.A., Malik M.H., Shahid S.A., Hayat A. (2017). Biomimetic nitrogen doped titania nanoparticles as a colorimetric platform for hydrogen peroxide detection. J. Colloid Interface Sci..

[27] Ahtzaz S., Nasir M., Shahzadi L., Amir W., Anjum A., Arshad R., Iqbal F., Chaudhry A.A., Yar M., ur Rehman I. (2017). A study on the effect of zinc oxide and zinc peroxide nanoparticles to enhance angiogenesis-pro-angiogenic grafts for tissue regeneration applications. Mater. Des..

[28] Shatnawi M., Alsmadi A., Bsoul I., Salameh B., Mathai M., Alnawashi G., Alzoubi G.M., Al-Dweri F., Bawa’aneh M. (2016). Influence of Mn doping on the magnetic and optical properties of ZnO nanocrystalline particles. Results Phys..

[29] Main K. (2017). Development of Composites for Bone Repair. Ph.D. Thesis.

[30] Buruiana T., Melinte V., Aldea H., Pelin I.M., Buruiana E.C. (2016). A new fluorinated urethane dimethacrylate with carboxylic groups for use in dental adhesive compositions. Mater. Sci. Eng. C.

[31] Soares R., Carone C., Einloft S., Ligabue R., Monteiro W. (2014). Synthesis and characterization of waterborne polyurethane/ZnO composites. Polym. Bull..

[32] Nguyen T.V., Nguyen T.A., Dao P.H., Nguyen A.H., Do M.T. (2016). Effect of rutile titania dioxide nanoparticles on the mechanical property, thermal stability, weathering resistance and antibacterial property of styrene acrylic polyurethane coating. Adv. Nat. Sci. Nanosci. Nanotechnol..

[33] Quiñones-Galván J., Sandoval-Jiménez I., Tototzintle-Huitle H., Hernández-Hernández L., de Moure-Flores F., Hernández-Hernández A., Campos-González E., Guillén-Cervantes A., Zelaya-Angel O., Araiza-Ibarra J. (2013). Effect of precursor solution and annealing temperature on the physical properties of Sol–Gel-deposited ZnO thin films. Results Phys..

[34] Talam S., Karumuri S.R., Gunnam N. (2012). Synthesis, characterization, and spectroscopic properties of ZnO nanoparticles. Int. Sch. Res. Not..

[35] Onwubu S.C., Mdluli P.S., Singh S., Bharuth V. (2019). Remineralization Potential of a Modified Eggshell–Titanium Composite-Scanning Electron Microscope Study. Eur. J. Dent..

[36] Senthilkumar S., Sivakumar T. (2014). Green tea (*Camellia sinensis*) mediated synthesis of zinc oxide (ZnO) nanoparticles and studies on their antimicrobial activities. Int. J. Pharm. Pharm. Sci..

[37] Aydin Sevinç B., Hanley L. (2010). Antibacterial activity of dental composites containing zinc oxide nanoparticles. J. Biomed. Mater. Res. Part B Appl. Biomater. J..

[38] Allaker R.P., Memarzadeh K. (2014). Nanoparticles and the control of oral infections. Int. J. Antimicrob. Agents.

[39] Bukhari J.H., Khan A.S., Ijaz K., Zahid S., Chaudhry A.A., Kaleem M. (2021). Low-temperature flow-synthesis-assisted urethane-grafted zinc oxide-based dental composites: Physical, mechanical, and antibacterial responses. J. Mater. Sci. Mater. Med..

[40] Anaya-Esparza L.M., Villagrán-de la Mora Z., Ruvalcaba-Gómez J.M., Romero-Toledo R., Sandoval-Contreras T., Aguilera-Aguirre S., Montalvo-González E., Pérez-Larios A. (2020). Use of Titanium Dioxide (TiO_2_) Nanoparticles as Reinforcement Agent of Polysaccharide-Based Materials. Process.

[41] Sodagar A., Akhoundi M.S.A., Bahador A., Jalali Y.F., Behzadi Z., Elhaminejad F., Mirhashemi A.H. (2017). Effect of TiO_2_ nanoparticles incorporation on antibacterial properties and shear bond strength of dental composite used in Orthodontics. Dent. Press J. Orthod..

[42] Poomalai P., Varghese T.O., Siddaramaiah (2011). Thermomechanical Behaviour of Poly(methyl methacrylate)/Copoly(ether-ester) Blends. ISRN Mater. Sci..

[43] Sideridou I.D., Karabela M.M., Vouvoudi E. (2011). Physical properties of current dental nanohybrid and nanofill light-cured resin composites. Dent. Mater. Off. Publ. Acad. Dent. Mater..

[44] Gilman J.W., Awad W.H., Davis R.D., Shields J., Harris R.H., Davis C., Morgan A.B., Sutto T.E., Callahan J., Trulove P.C. (2002). Polymer/Layered Silicate Nanocomposites from Thermally Stable Trialkylimidazolium-Treated Montmorillonite. Chem. Mater..

[45] Khan A.S., Khan R.S., Khan M., Rehman I.U., Subramani K., Ahmed W. (2019). Incorporation of nanoparticles in glass ionomer cements: Clinical applications, properties, and future perspectives. Nanobiomaterials in Clinical Dentistry.

[46] Erickson K.L. (2007). Thermal Decomposition of Polymers in Nitrogen and in Air.

[47] Barclay C.W., Spence D., Laird W.R. (2005). Intra-oral temperatures during function. J. Oral Rehabil..

[48] Younas B., Khan A.S., Muzaffar D., Hussain I., Chaudhry A.A., Rehman I.U. (2013). In situ reaction kinetic analysis of dental restorative materials. EPJ Appl..

[49] Lohbauer U., Zinelis S., Rahiotis C., Petschelt A., Eliades G. (2009). The effect of resin composite pre-heating on monomer conversion and polymerization shrinkage. Dent. Mater. Off. Publ. Acad. Dent. Mater..

[50] Nayyer M., Zahid S., Hassan S.H., Mian S.A., Mehmood S., Khan H.A., Kaleem M., Zafar M.S., Khan A.S. (2018). Comparative abrasive wear resistance and surface analysis of dental resin-based materials. Eur. J. Dent..

[51] Bociong K., Szczesio A., Sokolowski K., Domarecka M., Sokolowski J., Krasowski M., Lukomska-Szymanska M. (2017). The Influence of Water Sorption of Dental Light-Cured Composites on Shrinkage Stress. Materials.

[52] Kasraei S., Azarsina M. (2012). Addition of silver nanoparticles reduces the wettability of methacrylate and silorane-based composites. Braz. Oral Res..

[53] Acosta-Torres L.S., Lopez-Marin L.M., Nunez-Anita R.E., Hernandez-Padron G., Castano V.M. (2011). Biocompatible metal-oxide nanoparticles: Nanotechnology improvement of conventional prosthetic acrylic resins. J. Nanomater..

