# Supplementary material for: Structural, Physical, and Mechanical Analysis of ZnO and TiO2 Nanoparticle-Reinforced Self-Adhesive Coating Restorative Material
Source: Materials (Basel). 2021 Dec 7;14(24):7507. doi: 10.3390/ma14247507 (PMC8707675; doi:10.3390/ma14247507)
Supplement: Supplementary file 1 [file materials-14-07507-s001.zip › materials-1415027-supplementary.pdf]

*materials*

MDPI

*Supplementary Materials*

# Structural, Physical, and Mechanical Analysis of ZnO and $TiO_2$ Nanoparticle-Reinforced Self-Adhesive Coating Restorative Material

**Qura Tul Ain Idrees [1], Nazish Gul [1], Muhammad Amber Fareed [2], Salman Aziz Mian [3], Danish Muzaffar [4], Muhammad Nasir [5], Aqif Anwar Chaudhry [5], Sultan Akhtar [6], Syed Zubairuddin Ahmed [7] and Abdul Samad Khan [7,*]**

[1] Department of Science of Dental Materials, Postgraduate Medical Institute, Lahore 54000, Pakistan; aineshahzad@gmail.com (Q.T.A.I.); gul.nazish029@gmail.com (N.G.)

[2] Adult Restorative Dentistry, Dental Biomaterials and Prosthodontics Oman Dental College, Muscat 116, Oman; mafareed@staff.odc.edu.om

[3] Department of Dental Materials, Institute of Dentistry, CMH Lahore Medical College, Lahore 54000, Pakistan; salman_aziz@cmhlahore.edu.pk

[4] Faculty of Dentistry, SEGi University, Kota Damansara, Petaling Jaya 47810, Malaysia; danish9181@hotmail.com

[5] Interdisciplinary Research Centre in Biomedical Materials, COMSATS University Islamabad, Lahore Campus, Lahore 54000, Pakistan; muhammadnasir@cuilahore.edu.pk (M.N.); aqifanwar@cuilahore.edu.pk (A.A.C.)

[6] Department of Biophysics, Institute for Research and Medical Consultations, Imam Abdulrahman Bin Faisal University, Dammam 31441, Saudi Arabia; suakhtar@iau.edu.sa

[7] Department of Restorative Dental Sciences, College of Dentistry, Imam Abdulrahman Bin Faisal University, Dammam 31441, Saudi Arabia; szahmed@iau.edu.sa

* Correspondence: akhan@iau.edu.sa; Tel.: +966-59-478-1075

**Citation:** Idrees, Q.T.A.; Gul, N.; Fareed, M.A.; Mian, S.A.; Muzaffar, D.; Nasir, M.; Chaudhry, A.A.; Akhtar, S.; Ahmed, S.Z.; Khan, A.S. Structural, Physical, and Mechanical Analysis of ZnO and $TiO_2$ Nanoparticle-Reinforced Self-Adhesive Coating Restorative Material. *Materials* **2021**, *14*, 7507. https://doi.org/10.3390/ma14247507

Academic Editors: Alexandra Vinagre, Ramos Joao Carlos and Ana Messias

Received: 24 September 2021
Accepted: 26 November 2021
Published: 7 December 2021

**Publisher's Note:** MDPI stays neutral with regard to jurisdictional claims in published maps and institutional affiliations.

**Copyright:** © 2021 by the authors. Licensee MDPI, Basel, Switzerland. This article is an open access article distributed under the terms and conditions of the Creative Commons Attribution (CC BY) license (http://creativecommons.org/licenses/by/4.0/).

## Morphological analysis

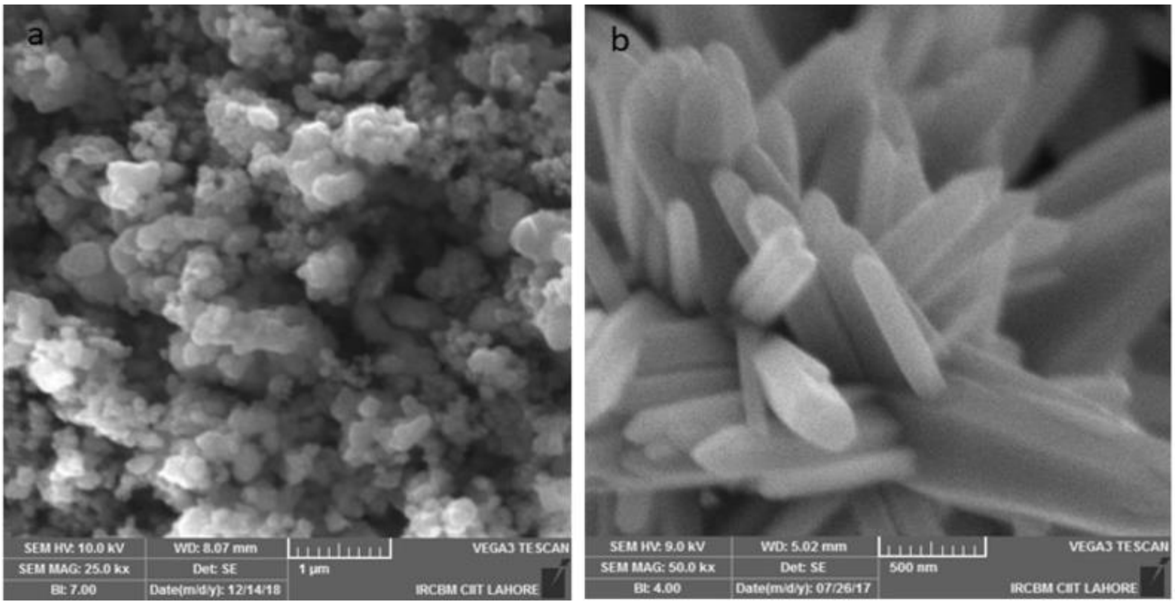


**Figure S1.** SEM images of (**a**) titanium dioxide and (**b**) zinc oxide nanoparticles, where the particle size of titanium dioxide and zinc oxide are 50-100 nm and 200-500 nm, respectively.

## Thermal analysis

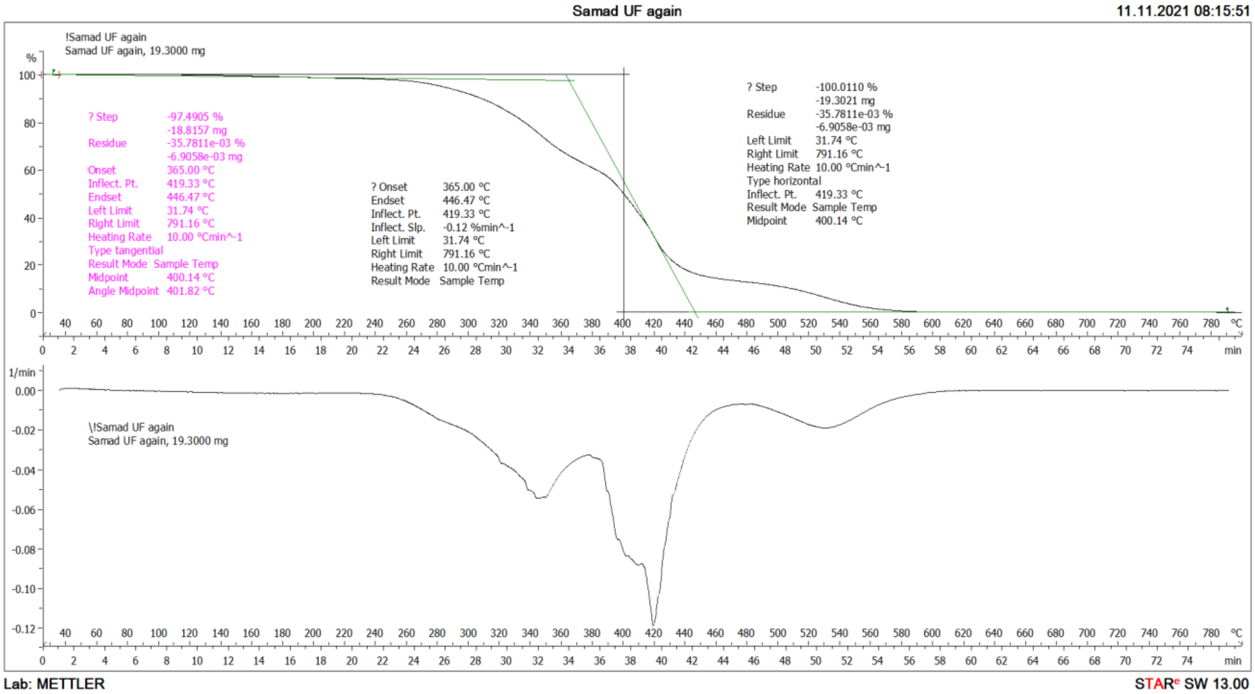


(a)

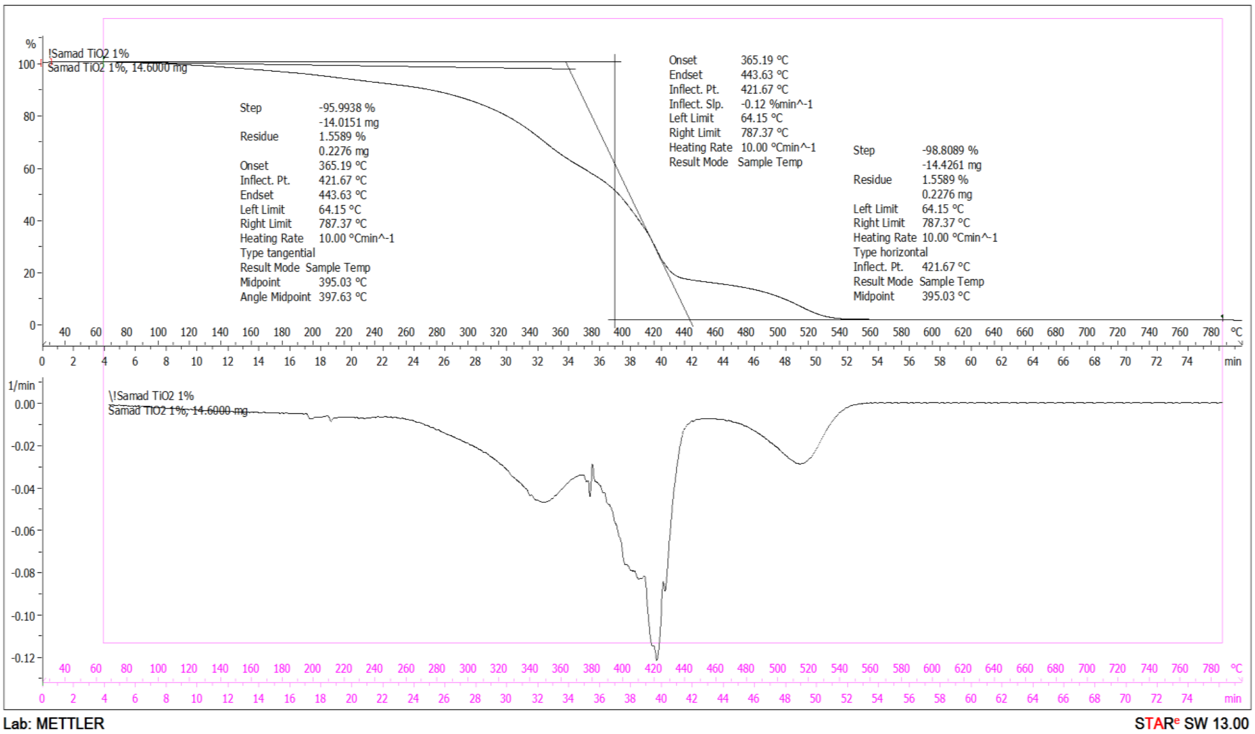
!Samad TiO2 1%
Samad TiO2 1%, 14.6000 mg
Step -95.9938 % -14.0151 mg
Residue 1.5589 % 0.2276 mg
Onset 365.19 °C
Inflect. Pt. 421.67 °C
Endset 443.63 °C
Left Limit 64.15 °C
Right Limit 787.37 °C
Heating Rate 10.00 °Cmin^-1
Type tangential
Result Mode Sample Temp
Midpoint 395.03 °C
Angle Midpoint 397.63 °C
Onset 365.19 °C
Endset 443.63 °C
Inflect. Pt. 421.67 °C
Inflect. Slp. -0.12 %min^-1
Left Limit 64.15 °C
Right Limit 787.37 °C
Heating Rate 10.00 °Cmin^-1
Result Mode Sample Temp
Step -98.8089 % -14.4261 mg
Residue 1.5589 % 0.2276 mg
Left Limit 64.15 °C
Right Limit 787.37 °C
Heating Rate 10.00 °Cmin^-1
Type horizontal
Inflect. Pt. 421.67 °C
Result Mode Sample Temp
Midpoint 395.03 °C
\!Samad TiO2 1%
Samad TiO2 1%, 14.6000 mg
%
1/min
°C
min
Lab: METTLER
STARe SW 13.00

(**b**)

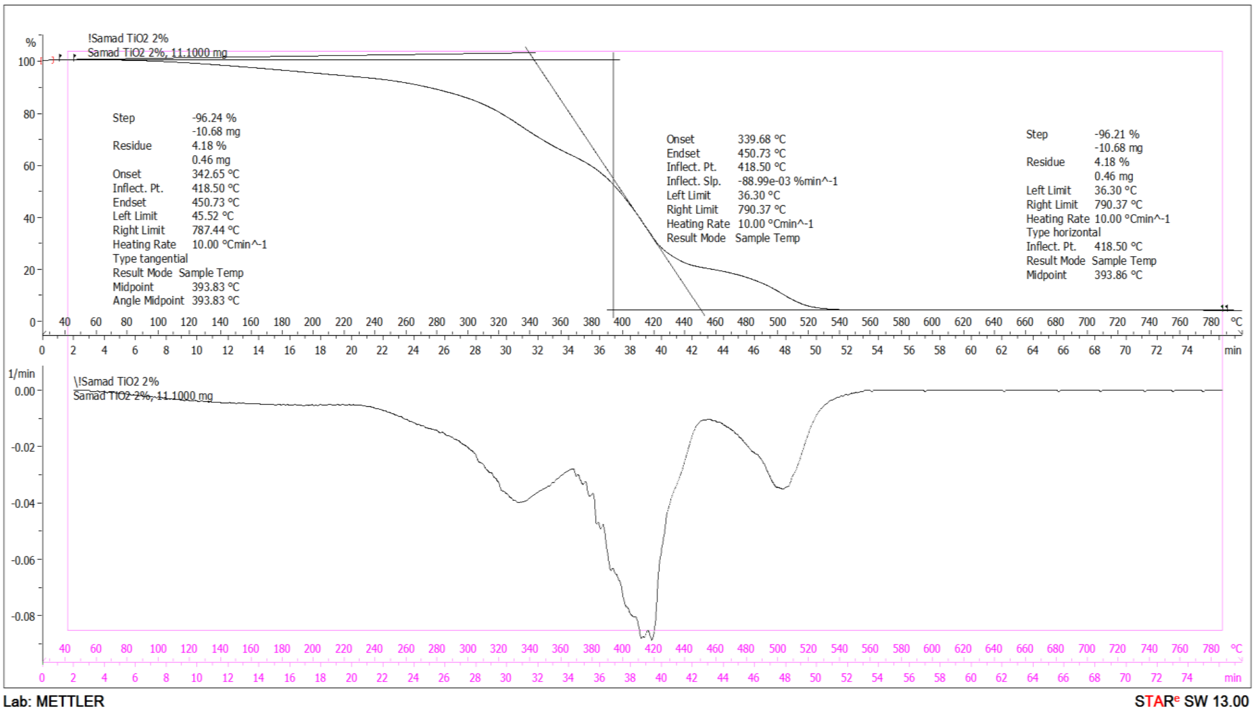
!Samad TiO2 2%
Samad TiO2 2%, 11.1000 mg
Step -96.24 % -10.68 mg
Residue 4.18 % 0.46 mg
Onset 342.65 °C
Inflect. Pt. 418.50 °C
Endset 450.73 °C
Left Limit 45.52 °C
Right Limit 787.44 °C
Heating Rate 10.00 °Cmin^-1
Type tangential
Result Mode Sample Temp
Midpoint 393.83 °C
Angle Midpoint 393.83 °C
Onset 339.68 °C
Endset 450.73 °C
Inflect. Pt. 418.50 °C
Inflect. Slp. -88.99e-03 %min^-1
Left Limit 36.30 °C
Right Limit 790.37 °C
Heating Rate 10.00 °Cmin^-1
Result Mode Sample Temp
Step -96.21 % -10.68 mg
Residue 4.18 % 0.46 mg
Left Limit 36.30 °C
Right Limit 790.37 °C
Heating Rate 10.00 °Cmin^-1
Type horizontal
Inflect. Pt. 418.50 °C
Result Mode Sample Temp
Midpoint 393.86 °C
\!Samad TiO2 2%
Samad TiO2 2%, 11.1000 mg
%
1/min
°C
min
Lab: METTLER
STARe SW 13.00

(**c**)

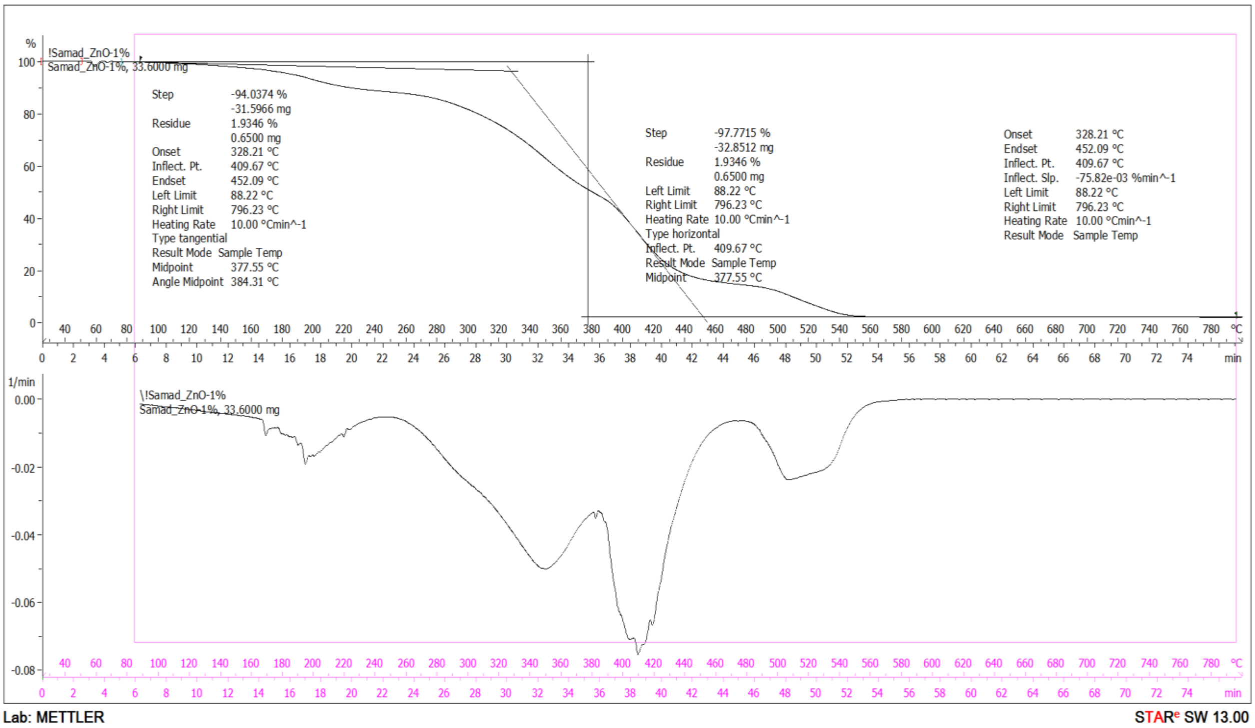


(**d**)

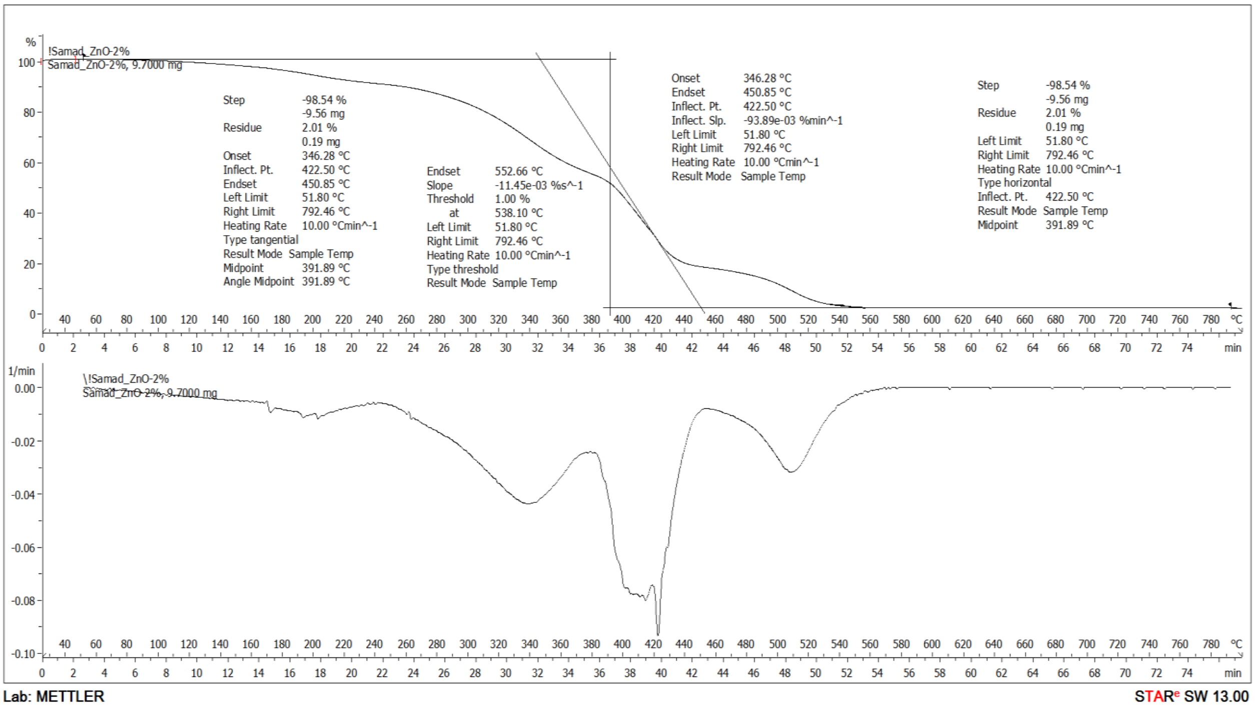


(**e**)

**Figure S2.** (**a**) Thermogram of EC-C showing weight loss (%) and derivative weight loss. (**b**) Thermogram of EC-T1 showing weight loss (%) and derivative weight loss. (**c**) Thermogram of EC-T2 showing weight loss (%) and derivative weight loss. (**d**) Thermogram of EC-Z1 showing weight loss (%) and derivative weight loss. (**e**) Thermogram of EC-Z2 showing weight loss (%) and derivative weight loss.
